# Twelve-Month Follow-Up of Dexamethasone Implants for Macular Edema from Various Diseases in Vitrectomized and Nonvitrectomized Eyes

**DOI:** 10.1155/2016/7984576

**Published:** 2016-09-18

**Authors:** Eduardo A. Novais, Mauricio Maia, Paulo Augusto de Arruda Mello Filho, João Rafael de Oliveira Dias, José Maurício B. B. Garcia, Gabriel C. de Andrade, Ricardo N. Louzada, Marcos Ávila, André Maia, J. Fernando Arevalo, Lihteh Wu, Maria Berrocal, Emmerson Badaró, Michel Farah

**Affiliations:** ^1^Department of Ophthalmology, Federal University of São Paulo, São Paulo, SP, Brazil; ^2^Brazilian Institute of Fighting Against Blindness (INBRACE), Assis/Presidente Prudente, SP, Brazil; ^3^Federal University of Goiás, Goiânia, GO, Brazil; ^4^Wilmer Eye Institute, Johns Hopkins University, Baltimore, MD, USA; ^5^Asociados de Macula Vitreo y Retina de Costa Rica, San Jose, Costa Rica; ^6^University of Puerto Rico, San Juan, PR, USA

## Abstract

*Purpose*. To evaluate the best-corrected visual acuity (BCVA), central retinal thickness (CRT), and the number of dexamethasone implants needed to treat cystoid macular edema (CME) from various etiologies over 12 months in vitrectomized and nonvitrectomized eyes.* Methods*. This multicenter retrospective cohort study included 112 patients with CME secondary to retinal diseases treated pro re nata (PRN) with a 0.7 mg intravitreal dexamethasone implant for 12 months. The BCVA, CRT, adverse events, safety data, and number of implants were recorded.* Results*. Vitrectomized and nonvitrectomized eyes received means of three implants and one implant, respectively, over 12 months (*P* < 0.001). The mean BCVA of all patients improved from 0.13 at baseline to 0.33 (*P* < 0.001) 12 months after one (*P* = 0.001), two (*P* = 0.041), and three (*P* < 0.001) implants but not four implants (*P* = 0.068). The mean baseline CRT decreased significantly (*P* < 0.001) from 463 to 254 microns after 12 months with one (*P* < 0.001), two (*P* = 0.002), and three (*P* = 0.001) implants but not with four implants (*P* = 0.114). The anatomic and functional outcomes were not significantly different between vitrectomized and nonvitrectomized eyes. Increased IOP was the most common adverse event (23.2%).* Conclusions*. Dexamethasone implant administered PRN improved VA and decreased CRT in CME, with possible long-term clinically relevant benefits for treating CME from various etiologies. Vitrectomized eyes needed more implants compared with nonvitrectomized eyes.

## 1. Introduction

Cystoid macular edema (CME) is an important cause of visual loss. It is usually related to vascular, infectious, and inflammatory conditions such as retinal vein occlusions (RVO), diabetic macular edema (DME), uveitis, postoperative macular edema, age-related macular degeneration, and radiation retinopathy [1]. Various factors and many presumed mechanisms can be involved in the pathogenesis of CME. These include mechanical traction, vascular endothelial growth factor- (VEGF-) induced CME, and inflammation [2]. In the past decade, novel therapeutic targets have shifted the treatment options from laser photocoagulation toward pharmacologic treatments. Of these, intravitreal anti-VEGF agents have been the most favored by most retina specialists. One of the main drawbacks of anti-VEGF therapy is its short duration of action which mandates monthly intravitreal injections in many patients [3].

Many inflammatory factors have been associated with CME of different etiologies. Intraocular corticosteroid therapies may decrease edema by inhibiting these factors and decreasing vascular permeability [2, 4]. The US Food and Drug Administration approved a dexamethasone intravitreal implant (Ozurdex®, Allergan, Inc., Irvine, CA) for treating CME associated with noninfectious posterior uveitis, RVO, and DME. The sustained-release formulation was designed to release dexamethasone from the implant for up to 6 months, thus controlling the inflammation for a longer time without the need for monthly injections [5]. However, steroid-related adverse events exist such as cataract formation and intraocular pressure (IOP) increases. Injection-related adverse events include retinal detachment and endophthalmitis. Pharmacokinetic studies have suggested that drug half-lives are shortened in vitrectomized eyes due to a more rapid drug clearance compared to nonvitrectomized eyes [6]. Other investigators have reported good results of the dexamethasone intravitreal implant in this set of eyes [7, 8]. The current study compared the functional and anatomic outcomes, adverse events, and number of intravitreal injections of the dexamethasone intravitreal implant between vitrectomized and nonvitrectomized eyes during a 12-month follow-up period.

## 2. Methods

This study was a multicenter, retrospective, open-label, exploratory chart review of data collected from patients with CME treated with one or more 0.7 mg dexamethasone implants at eight Latin American retina practices. The local institutional review board of Universidade Federal de São Paulo approved the study protocol, which adhered to the tenets of the Declaration of Helsinki.

### 2.1. Patient Selection

The inclusion criteria included eyes with a retinal disease associated with CME in the study eye(s), the absence of infectious uveitis as a cause of the CME, consent to the off-label use of the implant, implantation of at least one dexamethasone intravitreal implant, a minimal of 12-month follow-up after the first injection, and no other intravitreal injection of any drug for at least 6 months before the dexamethasone implant.

The exclusion criteria included patients with an incomplete chart information during the follow-up period; patient inability to complete the 12-month follow-up for any reason; a history of glaucoma or infectious uveitis/retinitis; and a previous intravitreal therapy such as Iluvien (pSivida, Watertown, MA), Retisert (pSivida), or Durasert (pSivida).

### 2.2. Medical Chart Information

Data were collected from the patient charts for monthly follow-up visits after implantation. The data included gender, age, ocular history, symptom duration, previous surgeries, IOP, best-corrected visual acuity (BCVA), baseline and 12-month fluorescein angiography (FA) images, baseline and monthly OCT findings after intraocular injection of the dexamethasone implant, and the number of dexamethasone implants needed to achieve complete CME regression during the 12-month period. Any adverse reactions related to the treatment, that is, increased IOP and lens opacity, and ocular procedures performed after dexamethasone implantation, that is, vitrectomy and glaucoma or cataract surgery, were recorded for statistical analysis.

### 2.3. Safety Analysis

The safety analysis assessed the changes in lens status, IOP, and injection-related adverse effects. Other secondary treatment-related adverse effects of the implants, such as cataract surgery due to lens opacification, need for topical IOP-lowering medications, and glaucoma surgery due to uncontrolled high IOP were also recorded.

### 2.4. Retreatment Criteria and Efficacy Analysis

The PRN regimen was used, and retreatment was considered when there was a clinically significant decrease of 10 letters in BCVA compared with baseline, a clinically significant increase of 50 *μ*m in central retinal thickness (CRT) compared with baseline, or a failure of the dexamethasone implant to produce a complete resolution of the CME after a total of two-month follow-up.

The drug efficacy was measured by calculating the mean change in the CRT based on the OCT findings and the Snellen BCVA measurements from baseline to the last follow-up visit compared to the number of dexamethasone implants needed to achieve complete CME regression.

### 2.5. Data Analysis and Statistical Methods

Continuous variables were summarized using descriptive statistics, including the sample size, mean, standard error, median, minimum, and maximum values. Categorical variables were summarized in frequency and percentage tables. The mean changes in the BCVA and CRT analyses included patients with a dexamethasone implant and 12-month follow-up; the 95% confidence intervals and statistical significance were analyzed using a generalized estimating equation model with a correlation structure. The nature and frequency of adverse events were tabulated throughout the study and summarized using descriptive statistics. Statistical analyses were evaluated suing the Mann-Whitney and Kruskal-Wallis tests. *P* < 0.05 was considered statistically significant.

## 3. Results

A total of 112 eyes of 112 patients (62 men (55%), 50 women (45%)) with a mean age of 64.5 ± 12.2 years (range, 19–88) were included. Retreatment was needed for patients with persistent or recurrent CME visualized on OCT images.  Table 1 shows the patient demographic data.

Retinal disease subgroups with a sufficient number of study eyes for meaningful analysis of functional and anatomic outcomes included DME (*n* = 43 eyes), BRVO (*n* = 17 eyes), CRVO (*n* = 14 eyes), and Irvine-Gass (*n* = 10 eyes). The groups with other etiologies were too small for separate analysis but were combined in the group that included all of the study eyes.  Table 2 shows the different etiologies of the CME.

The dexamethasone implant failed to achieve a complete resolution of the CME after a total of two-month follow-up in sixty patients (54%). Persistent leakage on fluorescein angiography at 12-month follow-up was present in seventy patients (63%).

### 3.1. Number of Implants

The mean number of treatments over the 12-month period was 1.35 (range, 1–5) in the entire study population. During the 12 months, 69 (61.6%) eyes needed one implant, 22 (19.6%) eyes two implants, 16 (14.3%) eyes three implants, four eyes (3.6%) four implants, and one eye (0.9%) five implants. The mean dexamethasone retreatment time was 6 months (range, 2–12 months). The mean number of implants in vitrectomized eyes during 12 months was three (range, 1–5), whereas nonvitrectomized eyes received one implant (range, 1–3), a difference that reached statistical significance (*P* < 0.001) (Table 3).

### 3.2. Snellen BCVA Analysis

The mean baseline BCVA was 0.13 in the entire study population; it improved significantly to 0.33 12 months after the first implant (*P* < 0.001). A separate analysis comparing the baseline with the 12-month follow-up BCVA related to the number of implants showed significant improvement in patients who received one (*P* = 0.001), two (*P* = 0.041), and three (*P* < 0.001) dexamethasone implants; however, injection of four implants did not result in a statistically significant (*P* = 0.068) difference (Table 4). The mean baseline BCVA in the nonvitrectomized eyes was 0.20 and in the vitrectomized eyes was 0.10 (*P* = 0.282). The 12-month mean BCVA in the nonvitrectomized eyes was 0.33 and in the vitrectomized eyes was 0.45, and this difference was not statistically significant (*P* = 0.418) (Table 3).

A subgroup analysis of the causes of CME showed statistically significant differences from baseline to 12 months. There were no differences among the diseases at baseline (*P* = 0.215); however, a statistically significant difference was noticed at the 12-month period (*P* = 0.007) (Table 5).

### 3.3. CRT

The mean baseline CRT measured on OCT images decreased significantly (*P* < 0.001) from 463 microns (range, 214–920 microns) to 254 microns (range, 154–977 microns) after 12 months of follow-up. Comparison of the baseline CRT with the 12-month follow-up CRT regarding the number of implants showed statistically significant improvements associated with injection of one (*P* < 0.001), two (*P* = 0.002), and three (*P* = 0.001) dexamethasone implants but not with four implants (*P* = 0.114) (Table 6). The mean baseline CRT in the nonvitrectomized eyes was 420 microns (range, 243–920 microns) and in the vitrectomized eyes was 532 microns (range, 214–840) (*P* = 0.199). The 12-month mean CRT in the nonvitrectomized eyes was 265 microns (range, 194–977 microns) and in the vitrectomized eyes was 232 microns (range, 154–741 microns), and this difference was not statistically significant (*P* = 0.3) (Table 3).

A subgroup analysis of the causes of ME showed statistically significant differences from baseline to 12 months, with no difference among the diseases (Table 5).

### 3.4. Adverse Events

Twenty-seven treatment-related adverse events were reported after the first injection. The most commonly reported drug-induced adverse event was an increased IOP, with a total of 26 (23.2%) events in the total population. All patients with this complication were treated successfully with a topical medication and no IOP-lowering surgery was required. The development of cataracts was another treatment-related adverse event that occurred in one patient (0.9%). In the current study, no injection-related adverse event such as endophthalmitis, retinal detachment, or ocular hypotony occurred.

## 4. Discussion

Anti-VEGF therapies remain the first-line option for treating CME associated with retinal diseases. A significant proportion of patients who either do not respond optimally to anti-VEGF therapy or have recurrent disease require frequent injections, which can become a substantial burden. The current study reports the results of using 0.7 mg dexamethasone intravitreal implants administered PRN at eight Latin American retina practices. All patients had VA that failed to improve and CME that failed to resolve resulting from a variety of retinal diseases in response to the current anti-VEGF and other therapies.

In the current study, injection of the dexamethasone implant resulted in significant improvements in BCVA and CRT, which is consistent with the results of large studies (MEAD and GENEVA) [9, 10]. In another retrospective German study in which 102 patients with RVO received one dexamethasone implant, significant improvements in BCVA and reductions in CRT were also observed [11]. Regarding other ocular indications, a small retrospective study of 27 patients with noninfectious uveitis reported that with repeated dexamethasone implant injections the retinal thickness improved and the inflammation resolved, which resulted in improved ocular function [12]. Functional and anatomic improvements after intravitreous implants have also been reported in several case series of patients with persistent DME [13–15].

In a similar study, Lam and associates [16] reported that the mean number of injections to treat CME from different causes was 1.7 ± 0.2, which was similar to our mean number of dexamethasone implants of 1.35 (range, 1–5). Those authors also showed that study eyes with uveitis had a better outcome with the dexamethasone implant compared to the eyes with other causes of CME. In our current study, however, no differences among the different diseases were identified. Another large retrospective study included 289 patients with RVO-related ME who received two or more intravitreous dexamethasone implants. The patients received a mean of 3.2 (range, 2–9) implants alone or combined with other therapies and the CRT and VA improved with each subsequent injection [17]. In the current study, when analyzing groups based on the numbers of injections, the gain in the BCVA and decrease in the CRT were not significant for patients who received four intravitreal dexamethasone implants. This may have been related to the disease severity and/or poor prognosis that required more aggressive treatment.

Clinicians frequently encounter patients with vitrectomized eyes who need intraocular injections. Many clinicians have speculated that the clearance of intravitreal anti-VEGF in vitrectomized eyes differs from that in nonvitrectomized eyes. The current literature is controversial regarding the impact of drug pharmacokinetics in vitrectomized eyes. In a rabbit model, Ahn and colleagues reported that the overall intraocular pharmacokinetic properties of ranibizumab (Lucentis, Genentech Inc., South San Francisco, CA) in vitrectomized eyes were similar to those in nonvitrectomized eyes [18]. In contrast, Christoforidis et al. [19] and Kakinoki et al. [20] showed that intravitreal half-life of bevacizumab (Avastin, Genentech Inc.) and ranibizumab decreased significantly after vitrectomy and lensectomy. Chin and associates reported that the concentration of intravitreal triamcinolone acetonide decreased more rapidly in the vitrectomized eye than in the nonvitrectomized eye, and therefore, faster clearance of intravitreal triamcinolone acetonide must be considered when planning intravitreal injections in vitrectomized eyes [21]. Pelegrín et al. recently compared dexamethasone intravitreal implants in vitrectomized and nonvitrectomized patients with noninfectious uveitis and reported a similar long-term safety profile and good response regarding decreased CRT and BCVA, with no significant differences in the numbers of reinjections [22]. In the current study, however, subgroup analysis showed that the mean numbers of injections in vitrectomized eyes during 12 months were significantly (*P* < 0.001) higher than in nonvitrectomized eyes over the same timeline to achieve the same anatomic and functional outcomes. This difference may have been due to the different diseases evaluated in the current study.

To the best of our knowledge, the current study was the first to compare the number of injections for dexamethasone implants in vitrectomized compared to nonvitrectomized eyes among different intraocular diseases and showed that the vitrectomized patients needed more injections based on the re-retreatment criteria in this retrospective series. Because the diseases and demographic data were similar between both groups (*P* > 0.05), we hypothesized that the half-life of a drug in the vitreous cavity of vitrectomized eyes was shorter and therefore the need for reinjections was higher.

Previous studies have also reported greater VA increases in pseudophakic eyes than in phakic eyes [9, 16]. The authors reported that these findings were secondary to cataract formation in phakic populations. Phakic patients are more prone to cataract development as an adverse effect of steroids especially in patients with diabetes [16, 23]. In the current study, repeated dexamethasone implant treatment could have increased the incidence of cataract development [24]. However, only one (0.9%) patient with diabetes developed a cataract during the 12-month period.

In addition to the one patient in whom a cataract developed, increased IOP developed in 26 (23.2%) patients, which was manageable using topical IOP-lowering medications without a glaucoma filtering surgery. The rate of ocular hypertension reported in this study is in agreement with the phase III trials of the dexamethasone implant in which, by the end of the study, no more than 24% of eyes with RVO and 23% of eyes with uveitis required IOP-lowering medications after treatment with the intravitreous implant [25, 26]. Seventy (63%) patients had FA leakage and 60 (54%) patients had persistence ME at the end of follow-up; however, these results did not agree with the significant VA improvement and decreased CRT possibly because some patients had only angiographic edema but no actually retinal thickening on OCT or partial resolution of the increased retinal thickness.

The major limitations of the current study were its retrospective and open-label design. No standardized assessments were defined before treatment across the centers, assessment tools such as OCT instruments were not normalized, and adverse events were limited to those reported in the medical charts. In addition, the patient data collected depended on the number of implants injected, frequency of treatment, and the length of the follow-up period. Analyses were limited to include data recorded from the patient charts; consequently, some extra information was not assessed.

In conclusion, the 0.7 mg dexamethasone implant improves VA and reduces the CRT in patients with CME secondary to various etiologies. Vitrectomized eyes appear to need more injections compared with nonvitrectomized ones. No important complications were observed in the current series. Further studies are needed to clarify the efficacy and safety of the dexamethasone implant in various patient populations in clinical settings.

## Figures and Tables

**Table 1:** Patient demographic data and the number of implants (*n* = 112 patients).

Female : male	50 (45%)/62 (55%)
Phakic/pseudophakic	31 (28%)/81 (72%)
Choroidal detachment	1 (0.9%)
Initial leakage on angiography	108 (96%)
Vitrectomized eyes	40 (36%)

Number of intravitreous implants	

1	69
2	22
3	16
4	4
5	1

**Table 2:** Causes of macular edema.

DME	43 (38%)
BRVO	17 (15%)
CRVO	14 (13%)
Irvine-Gass	10 (8.9%)
Age-related macular degeneration	7 (6.3%)
Epiretinal membrane	8 (7.1%)
CME in retinitis pigmentosa	4 (3.6%)
CME after primary pars plana vitrectomy for retinal detachment	4 (3.6%)
Uveitis	2 (1.8%)
CNV associated with central serous choroidopathy	1 (0.9%)
Pigment epithelial detachment	1 (0.9%)
CNV refractory to anti-VEGF in angioid streaks	1 (0.9%)

DME, diabetic macular edema; BRVO, branch retinal vein occlusion; CRVO, central retinal vein occlusion; CME, cystoid macular edema; CNV, choroidal neovascularization; VEGF, vascular endothelial growth factor.

**Table 3:** Baseline and 12-month BCVA and CRT related to the numbers of implants.

	Nonvitrectomized eyes (*n* = 72)	Vitrectomized eyes (*n* = 40)	*P* value^*∗*^
Baseline Snellen BCVA	0.20 (0.0–1.0)	0.10 (0.01–0.40)	0.282
12-month Snellen BCVA	0.33 (0.01–1.0)	0.45 (0.01–0.80)	0.418
Number of implants	1.0 (1.0–3.0)	3.0 (1.0–5.0)	<0.001
Baseline CRT (microns)	420 (243–920)	532 (214–840)	0.199
12-month CRT (microns)	265 (194–977)	232 (154–741)	0.300

^*∗*^By the Mann-Whitney test.

Data are expressed as the mean (minimum–maximum).

BCVA, best-corrected visual acuity; CRT, central retinal thickness.

**Table 4:** Baseline and 12-month BCVA related to the numbers of implants and vitrectomy.

	Baseline BCVA	12-month BCVA	*P* value
Number of implants			
All eyes	0.13 (0.00–1.00)	0.33 (0.01–1.00)	<0.001
1	0.20 (0.00–0.67)	0.33 (0.01–1.00)	0.001
2	0.20 (0.01–1.00)	0.50 (0.01–1.00)	0.041
3	0.10 (0.00–0.33)	0.33 (0.10–0.80)	<0.001
4	0.08 (0.01–0.20)	0.50 (0.20–0.80)	0.068

Vitrectomized			
No	0.20 (0.00–1.00)	0.33 (0.01–1.00)	0.001
Yes	0.11 (0.01–0.40)	0.45 (0.01–0.80)	<0.001

BCVA, best-corrected visual acuity.

Wilcoxon test.

**Table 5:** Subgroup analyses of baseline and 12-month BCVA levels and CRT on OCT images by disease.

	DME (*n* = 43 eyes)	BRVO (*n* = 17 eyes)	CRVO (*n* = 14 eyes)	Irvine-Gass (*n* = 10 eyes)	*P* value^*∗*^
BCVA					
Baseline Snellen VA	0.13 (0.01–0.67)	0.20 (0.00–1.00)	0.06 (0.01–0.50)	0.20 (0.13–0.67)	0.215
12-month Snellen VA	0.37 (0.01–1.00)	0.33 (0.03–0.80)	0.04 (0.01–0.67)	0.67 (0.67–1.00)	0.007

OCT					
Baseline CRT (*µ*m)	444 (214–746)	421 (302–674)	693 (415–840)	686 (313–777)	0.058
12-month CRT (*µ*m)	264 (164–702)	289 (203–741)	367 (213–700)	220 (216–244)	0.15

^*∗*^Kruskal-Wallis for independent samples.

Data are expressed as the mean (minimum–maximum).

OCT, optical coherence tomography; BCVA, best-corrected visual acuity; DME, diabetic macular edema; BRVO, branch retinal vein occlusion; CRVO, central retinal vein occlusion; CRT, central retinal thickness.

**Table 6:** Baseline and 12-month CRT related to numbers of implants and vitrectomy.

	Baseline CRT	12-month CRT	*P* value
Number of implants			
All eyes	463 (214–920)	254 (154–977)	<0.001
1	418 (244–920)	279 (196–977)	<0.001
2	469 (302–746)	270 (164–634)	0.002
3	487 (214–777)	232 (185–402)	0.001
4	733 (510–840)	242 (168–741)	0.144

Vitrectomized			
No	421 (243–920)	265 (194–977)	<0.001
Yes	532 (214–840)	232 (154–741)	<0.001

CRT, central retinal thickness.

Wilcoxon test.
